# Supplementary material for: A-kinase anchoring protein BIG3 coordinates oestrogen signalling in breast cancer cells
Source: Nat Commun. 2017 May 30;8:15427. doi: 10.1038/ncomms15427 (PMC5512694; doi:10.1038/ncomms15427)
Supplement: Supplementary Information — Supplementary Figures and Supplementary Tables [file ncomms15427-s1.pdf]

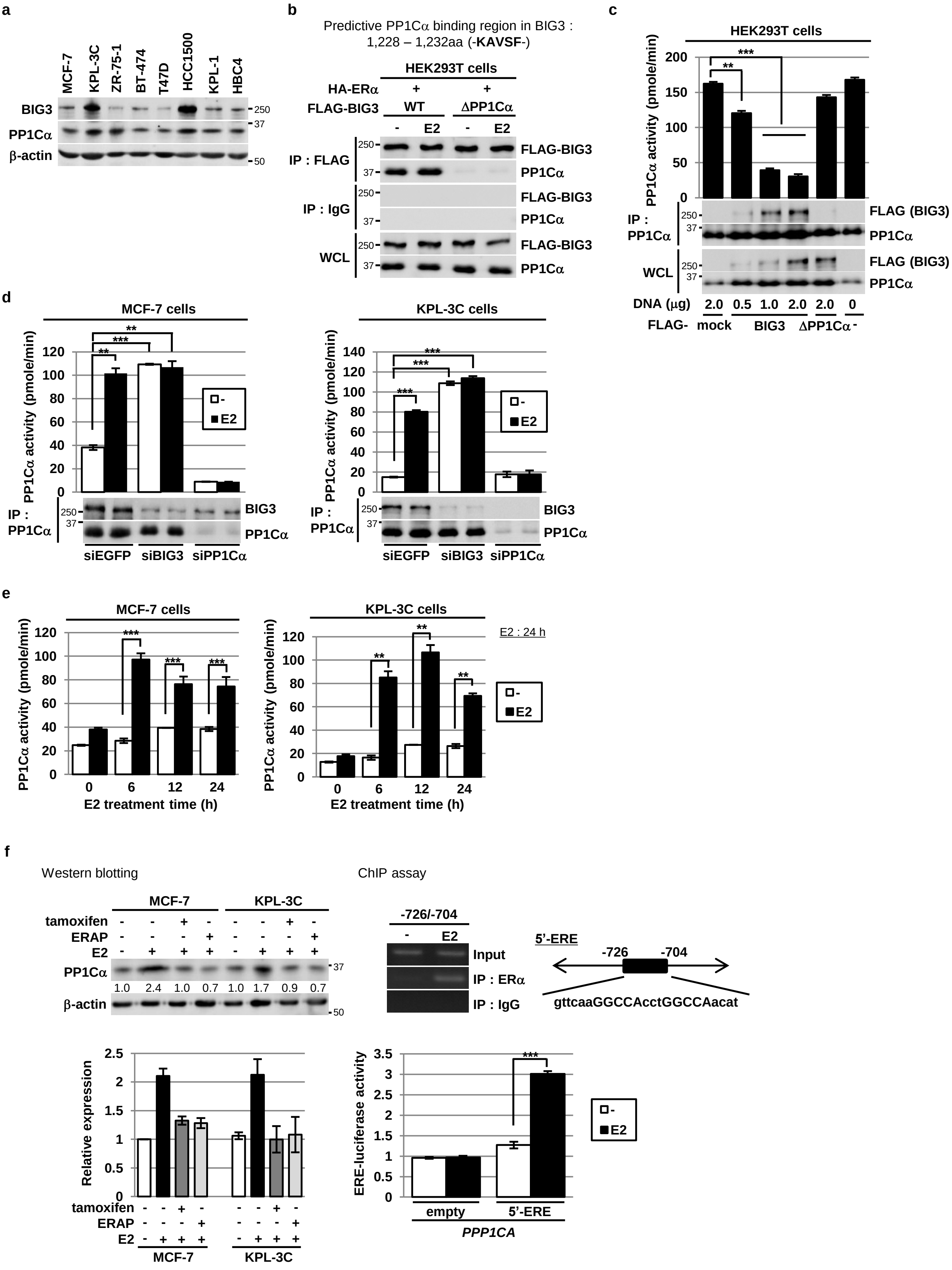
a
MCF-7
KPL-3C
ZR-75-1
BT-474
T47D
HCC1500
KPL-1
HBC4
BIG3
PP1Cα
β-actin
250
37
50
b
Predictive PP1Cα binding region in BIG3 :
1,228 – 1,232aa (-KAVSF-)
HEK293T cells
HA-ERα
FLAG-BIG3
WT
ΔPP1Cα
E2
IP : FLAG
IP : IgG
WCL
FLAG-BIG3
PP1Cα
c
HEK293T cells
PP1Cα activity (pmole/min)
IP : PP1Cα
WCL
FLAG (BIG3)
PP1Cα
DNA (μg)
2.0
0.5
1.0
2.0
2.0
0
FLAG-
mock
BIG3
ΔPP1Cα
d
MCF-7 cells
KPL-3C cells
PP1Cα activity (pmole/min)
E2
IP : PP1Cα
BIG3
PP1Cα
siEGFP
siBIG3
siPP1Cα
e
MCF-7 cells
KPL-3C cells
PP1Cα activity (pmole/min)
E2 : 24 h
E2 treatment time (h)
0
6
12
24
f
Western blotting
ChIP assay
MCF-7
KPL-3C
tamoxifen
ERAP
E2
PP1Cα
β-actin
1.0
2.4
1.0
0.7
1.0
1.7
0.9
0.7
-726/-704
Input
IP : ERα
IP : IgG
5'-ERE
-726
-704
gttcaaGGCCAcctGGCCAacat
Relative expression
ERE-luciferase activity
empty
5'-ERE
PPP1CA

g

Position	Sequence	PSSM Score
228 - 248	QLLYLECILSVLSSSSSSMHL	43.4
**318 - 338**	**VARTIYYIAAELVRLVGSVDS**	**24.2**
439 - 459	ELSQGKGLSEGQVQLLLLRLE	74.4
496 - 516	GNERSLDISISVTTDTGQTTL	26.2
1106 - 1126	SGSSAAKVVLTLSTQADRLFE	42.4
**1225 - 1245**	**VSQKAVSFIHDILTEVLTDWN**	**20.6**
1449 - 1469	GLIEVWIILLEQLTAAVSNCP	83.0

h

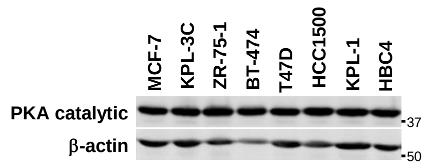

i

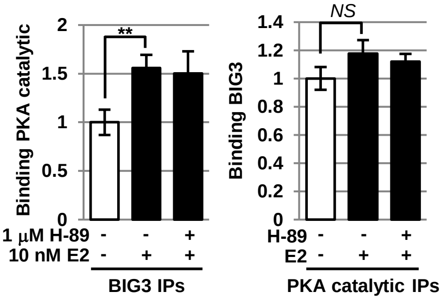

j

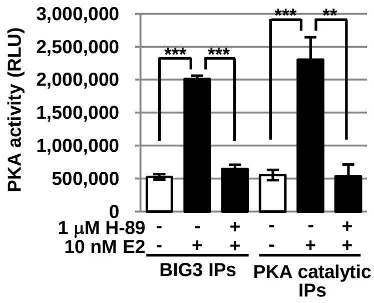

k

Target site	Score
T162	0.79
S305	0.79
S689	0.81
S925	0.83
S1208	0.78
S1763	0.80

(*Analysis by NetPhos 3.1*)

l

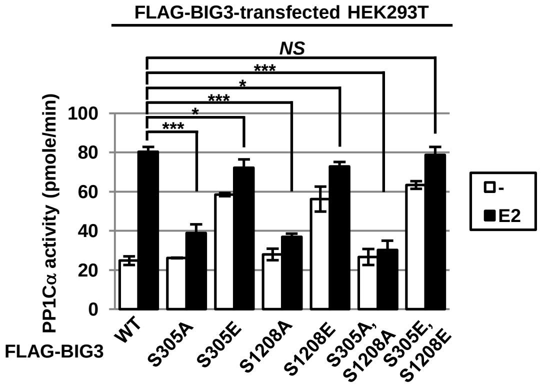

m

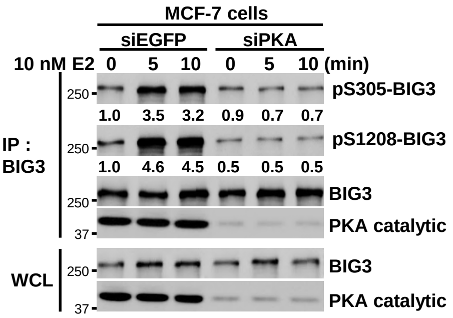

n

S305-Peptide (DHGRG**S**GCSCTAPALSGPVAR**)**
+ recombinant PKA for 30 min at 30˚C

Score	Sequence	Modification
83.14	GSGCSCTAPALSGPVAR	-
57.63	G**S**GCSCTAPALSGPVAR	2S: Phospho<br>4C: Carboxymethyl<br>6C: Propionamide

S1208-Peptide (RCW**S**LVAPH**)**
+ recombinant PKA for 30 min at 30˚C

Score	Sequence	Modification
72.15	CWSLVAPH	-
56.34	CW**S**LVAPH	1C: Propionamide<br>3S: Phospho

Supplementary Figure 1 | BIG3 regulated PP1Cα activity through its phosphorylation by PKA. (**a**) Expression patterns of BIG3 and PP1Cα in breast cancer cell lines. β-actin served as a quantitative internal control. (**b**) Identification of the predictive PP1Cα binding region in BIG3. The indicated FLAG-tagged BIG3 constructs (WT; full-length BIG3, ΔPP1Cα; BIG3 deleting PP1Cα-binding region, 1,228-KAVSF-1,232) and HA-tagged ERα construct transfected-HEK293T cells were immunoprecipitated using an anti-FLAG antibody. (**c**) The inhibitory effects of BIG3 overexpression on phosphatase activity of PP1Cα-immunoprecipitates in FLAG-tagged BIG3 or ΔPP1Cα construct-transfected HEK293T cells using *p*NPP as a substrate. These data represent the means ± s.e.m. of three independent experiments. (**d**) Effects of siPP1Cα and siBIG3 on phosphatase activity of PP1Cα immunoprecipitates using *p*NPP as a substrate in MCF-7 and KPL3C cells. These data represent the means ± s.e.m. of three independent experiments. (**e**) Phosphatase activity of PP1Cα after E2 stimulation in MCF-7 (left) and KPL-3C (right) cells. These data represent the means ± s.e.m. of three independent experiments. (**f**) Positive

feedback regulation of *PPP1CA* transactivation. Left, Effects of tamoxifen on *PPP1CA* expression. For immunoblot analysis (upper), β-actin served as a loading control. For real-time PCR analysis (lower), the data are expressed as the fold-increase over untreated cells (set at 1.0). These data represent the mean ± s.e.m. of three independent experiments. Right, ChIP assays of the transactivation of *PPP1CA* through an ERE motif in 5' upstream (upper), and luciferase assays of the transactivation of *PPP1CA* using a luciferase reporter containing an ERE motif conserved within 5' upstream of the *PPP1CA* gene (lower). The data represent the mean ± s.e.m. of three independent experiments. (**g**) The predicted PKA binding regions in BIG3, as determined using Hou *et al.*[24] and the PSIVER software. The bold letters indicate the potential PKA binding regions in BIG3. (**h**) Expression patterns of PKA protein in breast cancer cell lines. (**i**) Statistical analysis of PKA and BIG3 binding to BIG3 (right) and PKA (left) immunoprecipitates, respectively. These data are expressed as the fold-increase over untreated cells (set at 1.0), and represent the mean ± s.e.m. of three independent experiments. (**j**) Statistical analysis of *in vitro* PKA activity of BIG3 and PKA immunoprecipitates. These data represent the mean ± s.e.m. of three independent experiments. (**k**) The predicted phosphorylation sites of BIG3 by PKA, as determined using NetPhos 3.1 software. (**l**) Phosphatase activity of PP1Cα in a pseudo-phosphorylation mutant of BIG3 (S305E and S1208E) and alanine mutant of BIG3 (S305A and S1208A). (**m**) The inhibitory effects of siPKA on BIG3 phosphorylation. Representative results are shown from one of two experiments. (**n**) 2DICAL analysis of engineered peptides representing S305 and S1208 on BIG3 with recombinant PKA (recPKA). **P<0.01, ***P<0.001 (two-sided Student's *t*-test)

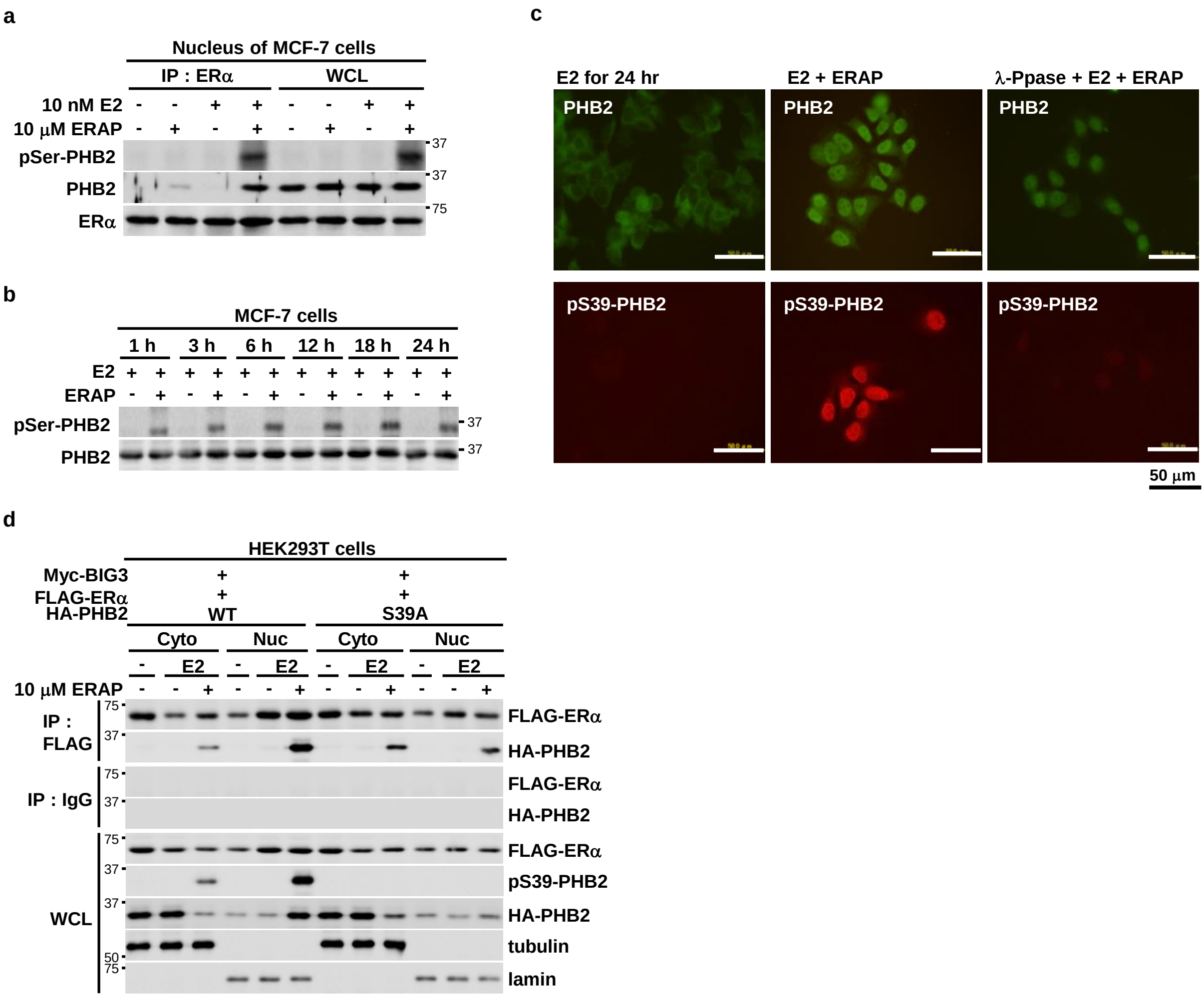

Supplementary Figure 2 | PHB2 is phosphorylated at S39 via PKCα. (**a**) Serine phosphorylation of PHB2 in ERα immunoprecipitates of the nuclear fraction of MCF-7 cells after E2 ± ERAP treatment for 24 h. (**b**) Time course of serine phosphorylation of PHB2 in MCF-7 cells after E2 ± ERAP treatment for the indicated time. (**c**) Representative immunofluorescence images of PHB2 phosphorylation at S39; PHB2 (green), phosphorylated S39 PHB2 (red). (**d**) The subcellular localization of PHB2 phosphorylation at S39 in the presence of E2 in the cytoplasmic (Cyto) and nuclear (Nuc) fractions of the indicated PHB2 (WT, S39A) and ERα construct-transfected HEK293T cells. a/b-tubulin (tublin) and laminin B (lamin) were used as loading controls for the cytoplasmic and nuclear fractions, respectively.

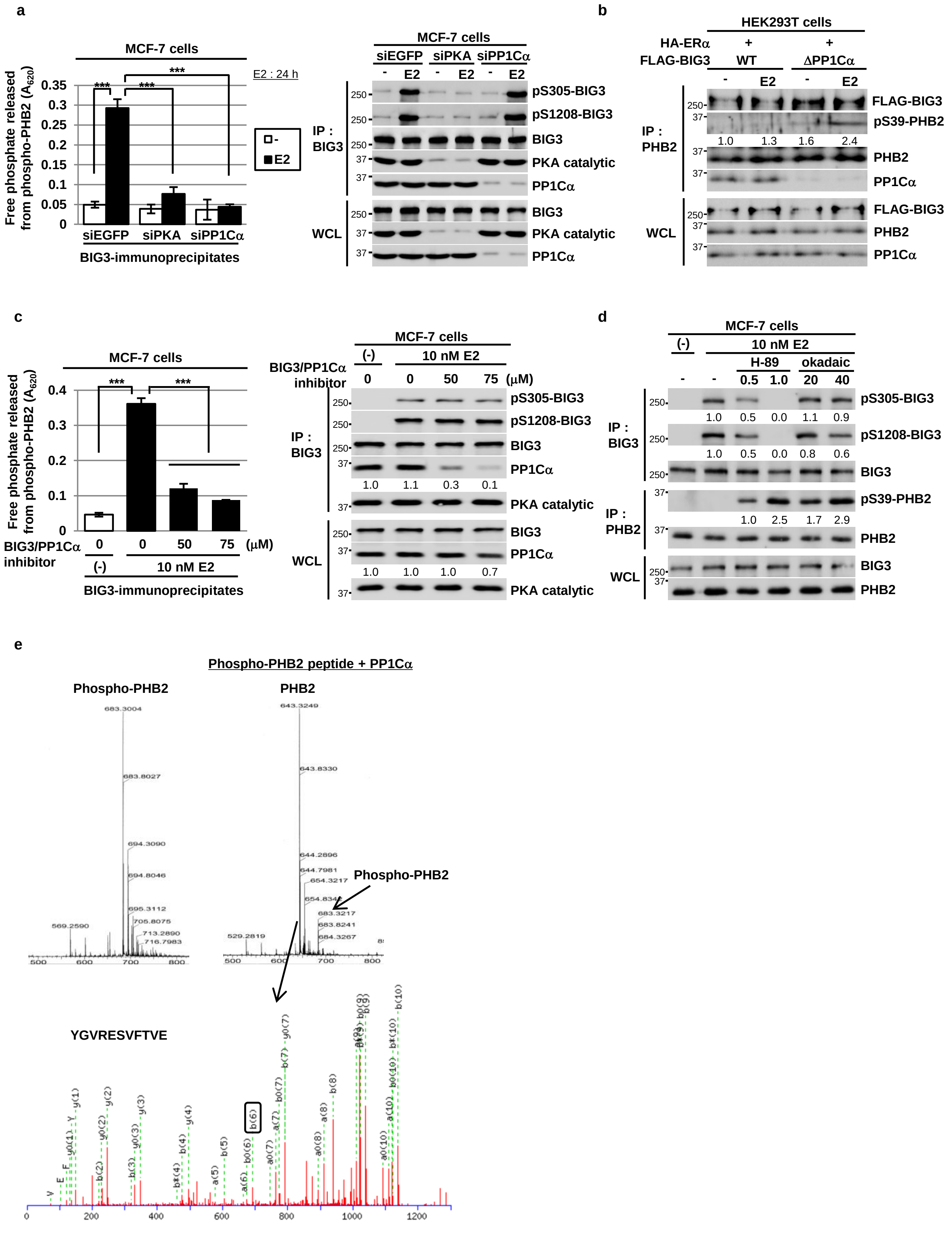

Supplementary Figure 3 | Phosphatase activity of BIG3-PP1Cα regulates PHB2 phosphorylation. (**a**) Phosphatase activity (left) and Western blot analyses (right) of BIG3 immunoprecipitates of PP1Cα- and PKA-depleted MCF-7 cells against phospho-S39 PHB2 peptide. Phosphatase activity was measured using malachite green. These data represent the means ± s.e.m. of three independent experiments. (**b**) The inhibitory effect of PP1Cα on PHB2 S39 phosphorylation. The indicated

FLAG-tagged BIG3 (WT, ΔPP1Cα) and HA-tagged ERα-transfected HEK293T cells, followed by immunoprecipitation with an anti-PHB2 antibody. (**c**) The inhibitory effect of BIG3-PP1Cα binding inhibitor on PP1Cα phosphatase activity. MCF-7 cells were treated with BIG3-PP1Cα binding inhibitor for 24 h in the presence of E2 and were immunoprecipitated using BIG3 antibody. (**d**) The effects of PKA inhibitor H-89 and PP1Cα inhibitor okadaic acid on BIG3 phosphorylation (S305 and S1208) and PHB2 phosphorylation (S39). MCF-7 cells were treated with H-89 or okadaic acid for 24 h in the presence of E2 and were immunoprecipitated using BIG3 or PHB2 antibody. (**e**) Q-TOF spectra indicating dephosphorylation of phospho-PHB2-peptide by PP1Cα in positive ion mode. The dephosphorylation of peptide is exemplarily shown for *m*/*z* 643.3. Upper, the left spectrum is an untreated control, and the right spectrum was acquired after dephosphorylation with PP1Cα. Lower, product ion spectrum of the PHB2 peptide YGVRESVFTVE, with a precursor mass of *m*/*z* 643.3. *** $P<0.001$ (two-sided Student's *t*-test).

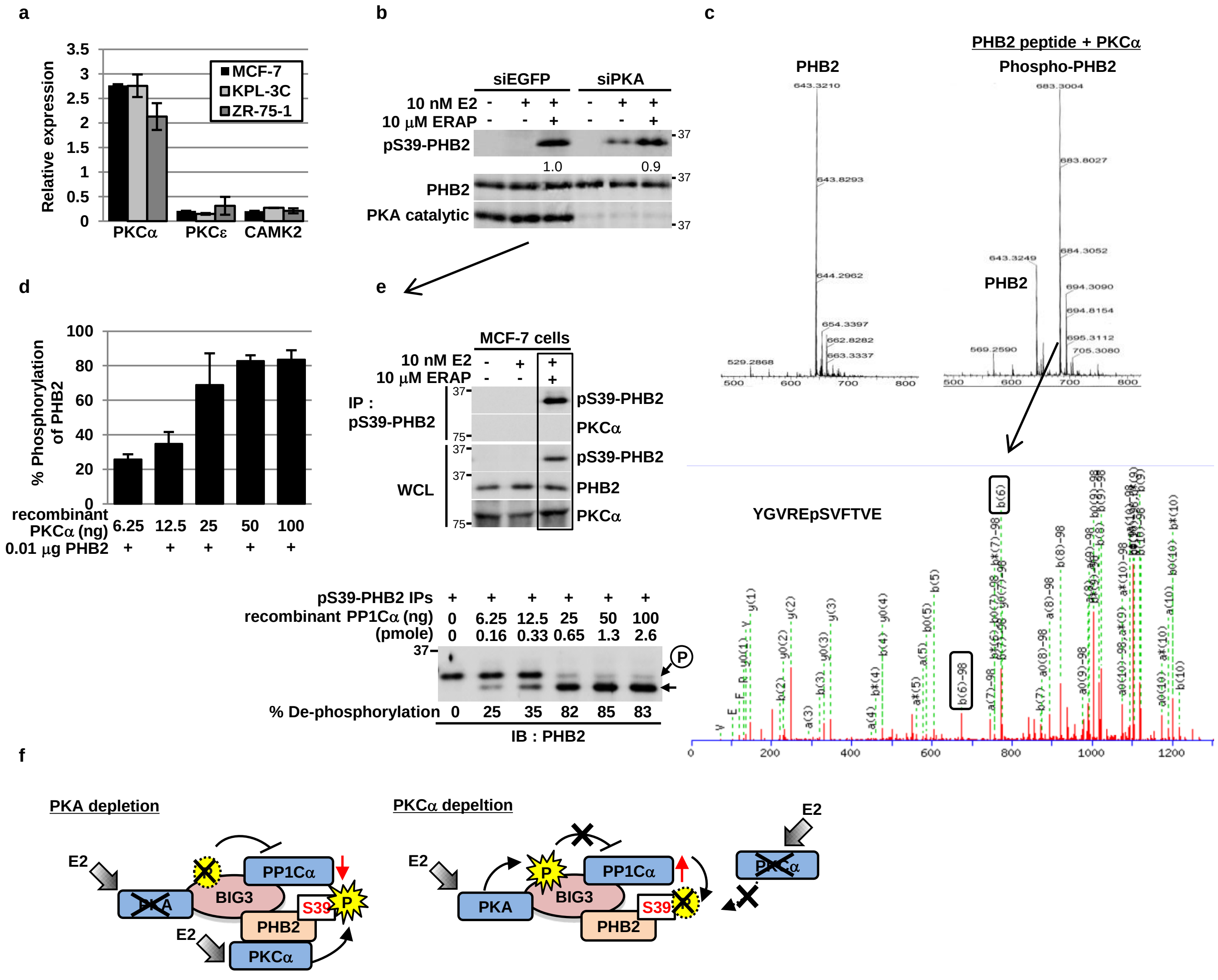

Supplementary Figure 4 | PKCα is confirmed to be responsible kinase for PHB2 S39 phosphorylation. (**a**) Expression patterns of *PKCα, PKCε*, and *CAMK2* in ERα-positive breast cancer cell lines (MCF-7, KPL-3C and ZR-75-1) using real-time PCR. (**b**) The inhibitory effects of siPKA on PHB2 phosphorylation at S39 in MCF-7 cells after E2 ± ERAP treatment for 24 h. (**c**) Q-TOF spectra of direct PHB2 peptide phosphorylation by PKCα in positive ion mode. The phosphorylation of peptide is exemplarily shown for *m/z* 683.3. Upper, the left spectrum is an untreated control, and the right spectrum was acquired after phosphorylation with PKCα. Lower, product ion spectrum of the phosphor-S39 PHB2 peptide YGVRE(pS)VFTVE, with a precursor mass of *m/z* 683.3. (**d**) Statistical analysis of the ratio of phosphorylation at S39 in full-length PHB2 by PKCα. These data are expressed as the percentage of phosphorylated band in total PHB2 band and represent the mean ± s.e.m. of four independent experiments. (**e**) Dephosphorylation of PHB2 phosphorylation at S39 immunoprecipitated by phospho-specific PHB2 (S39) antibody from MCF-7 cells treated with E2/ERAP for 24 h. (**f**) Schematic illustration of BIG3-PKA-PP1Cα tri-complex under the depletion of PKA (left) and PKCα (right).

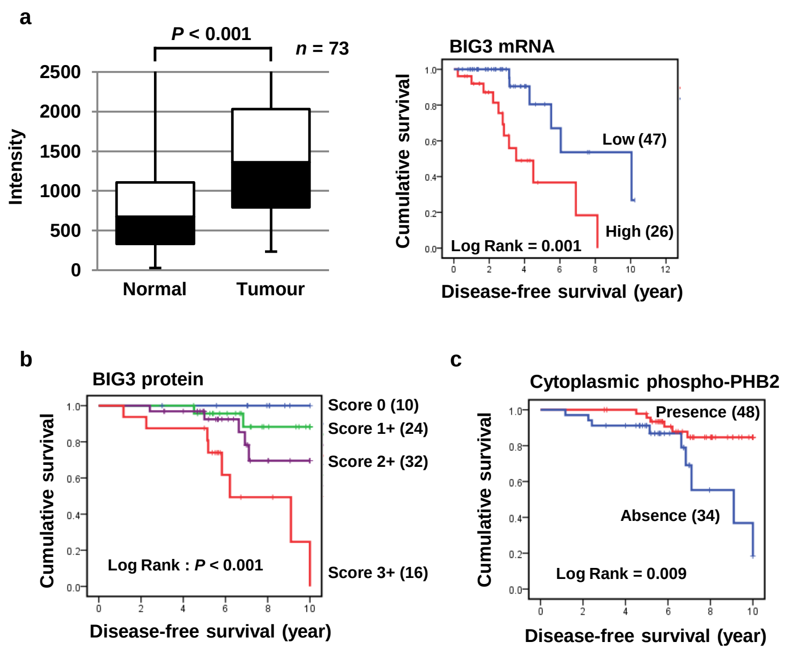

Supplementary Figure 5 | BIG3 overexpression was predictive of worse outcomes in ERα-positive breast cancer. (**a**) mRNA expression of *BIG3* in patients with ERα-positive breast cancer (left) and Kaplan-Meier curves of overall survival as a function of *BIG3* expression (right) based on the TCGA data set. (**b**, **c**) Kaplan-Meier analysis of survival associated with BIG3 protein (**b**) and cytoplasmic PHB2 phosphorylation at S39 (**c**) in representative ERα-positive breast cancer specimens.

Uncropped images of Figure 1a

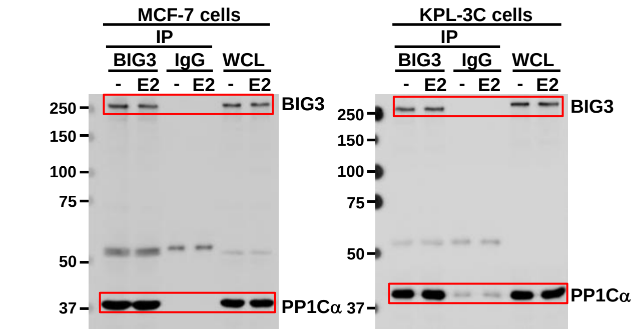

Uncropped images of Figure 1b

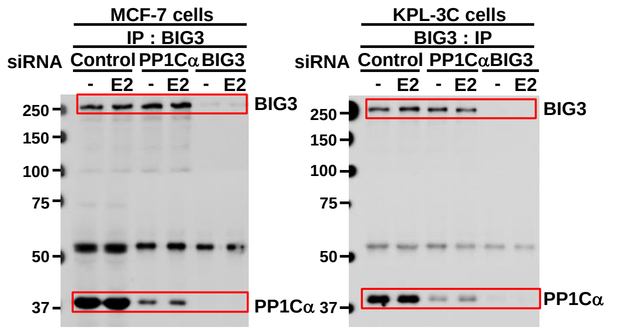

Uncropped images of Figure 1c

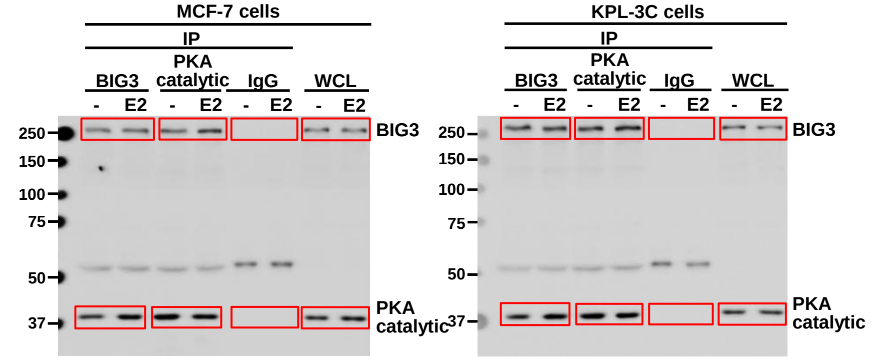

Uncropped images of Figure 1d

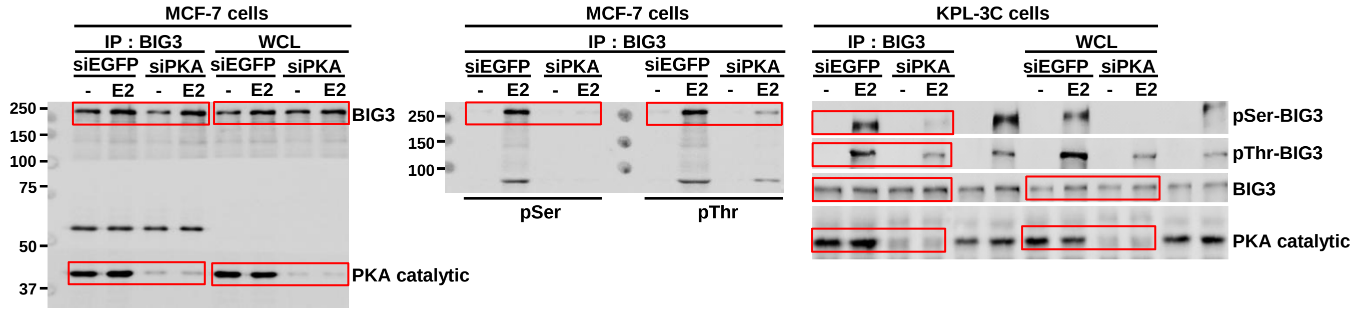

Uncropped images of Figure 1f

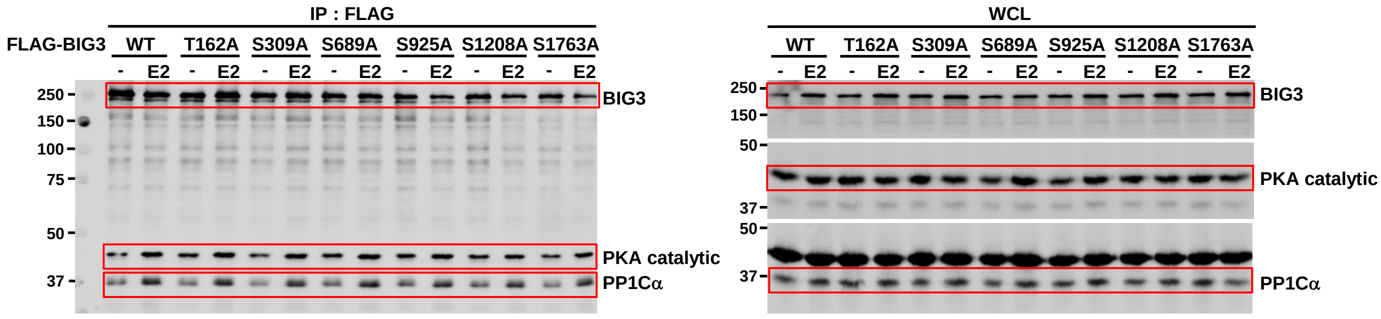

Continued on the following page

Uncropped images of Figure 1g

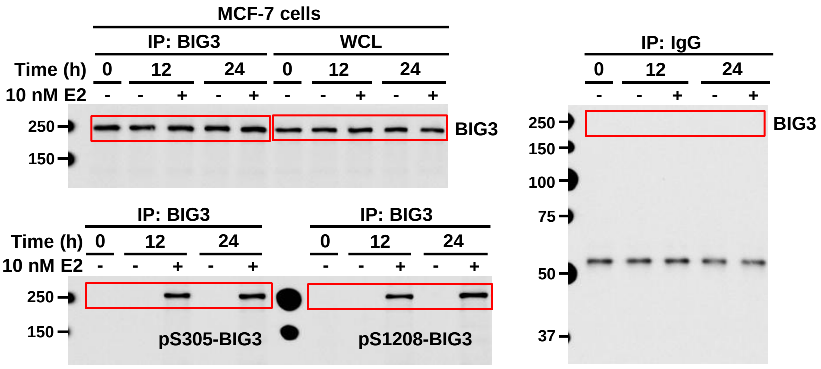

Uncropped images of Figure 2b

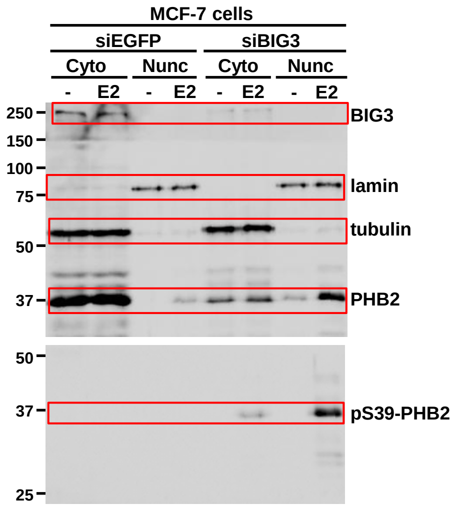

Uncropped images of Figure 2c

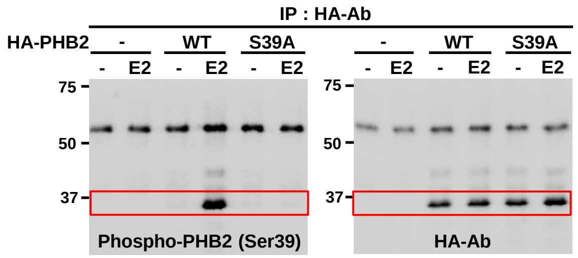

Uncropped images of Figure 2d

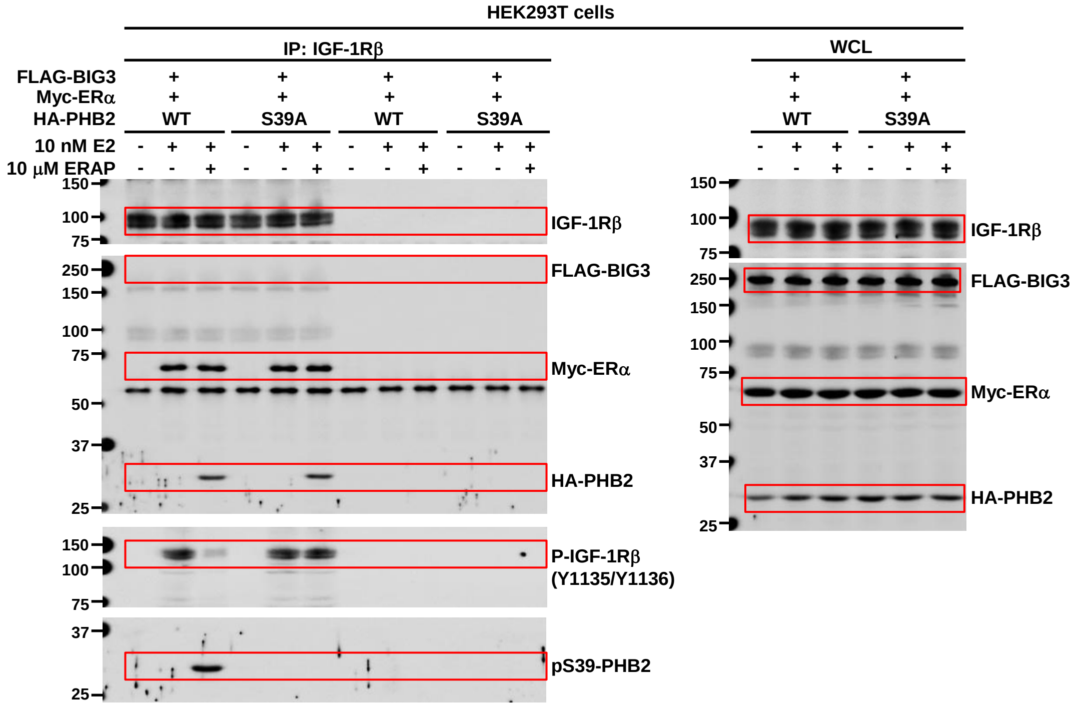

Continued on the following page

Uncropped images of Figure 2e

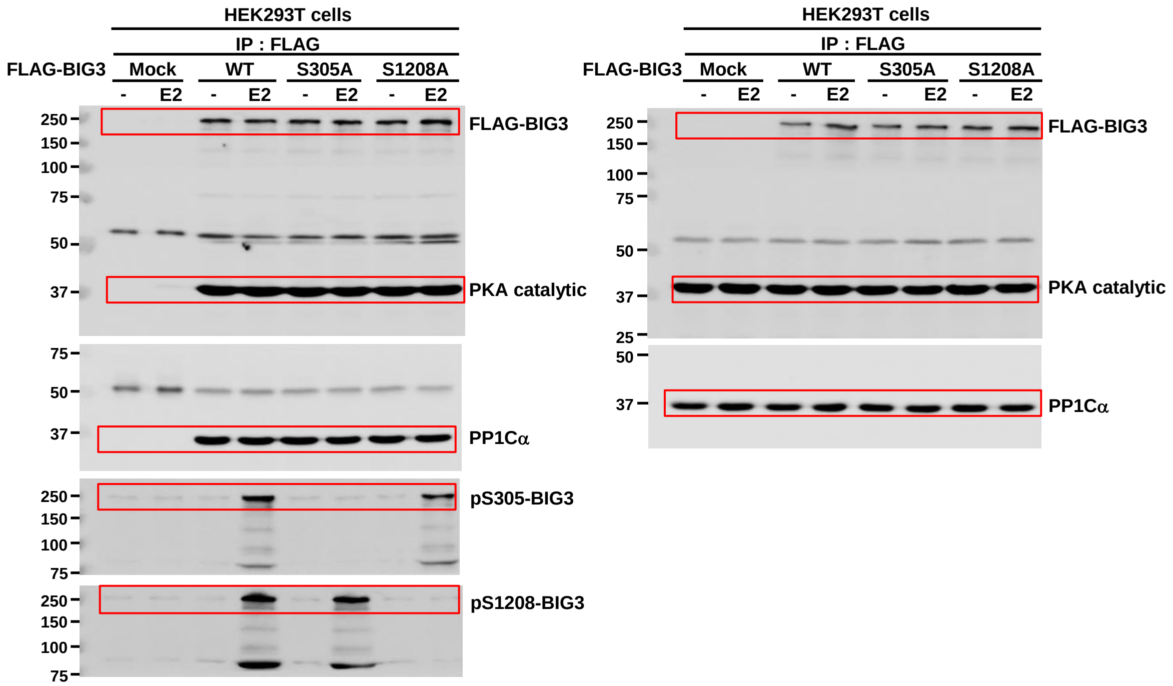

Uncropped images of Figure 2f

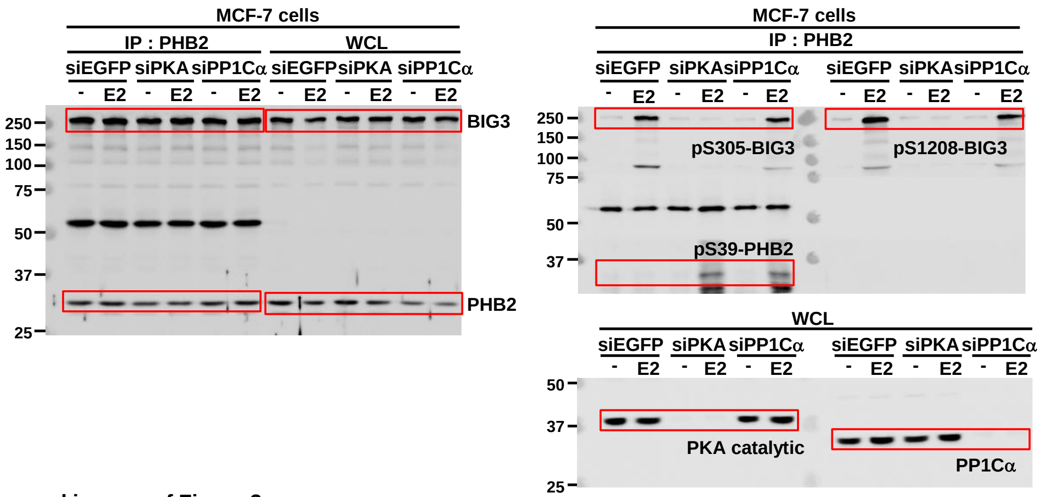

Uncropped images of Figure 3a

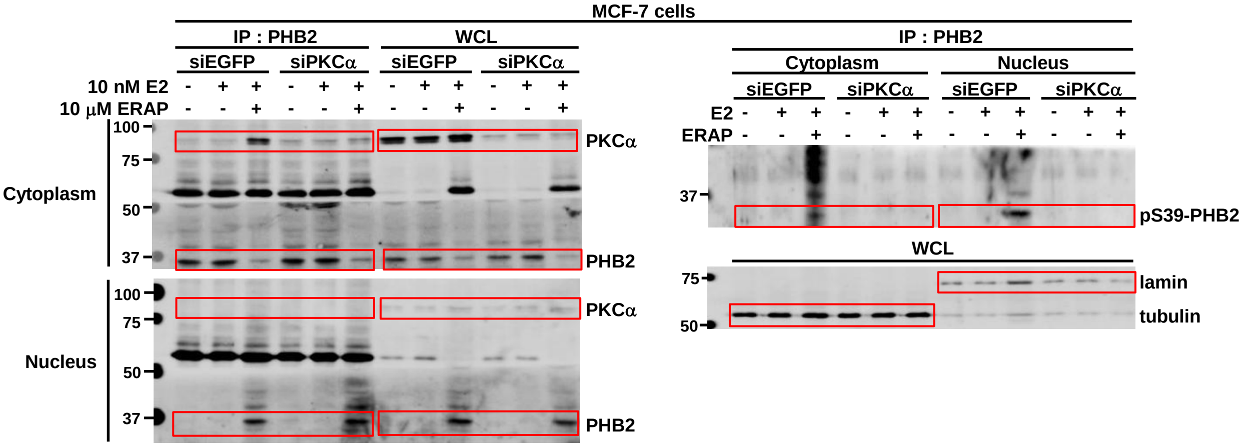

Continued on the following page

Uncropped images of Figure 3c

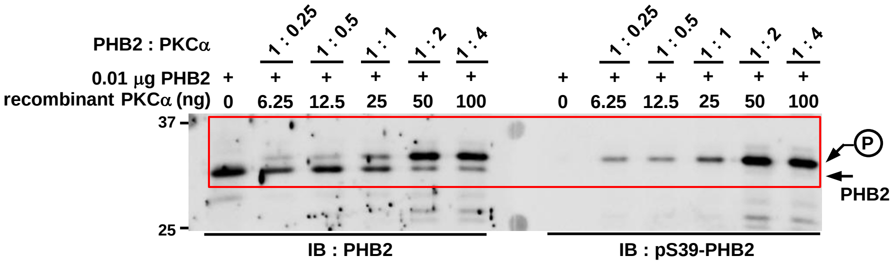

Uncropped images of Figure 3d

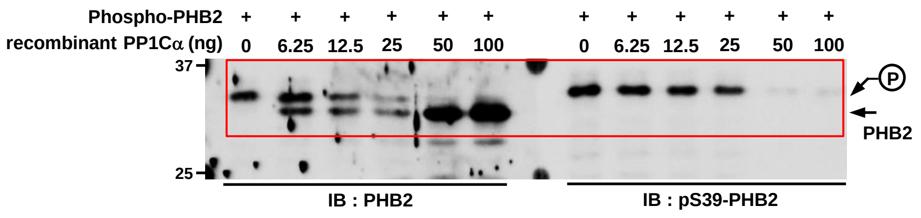

Uncropped images of Figure 3e

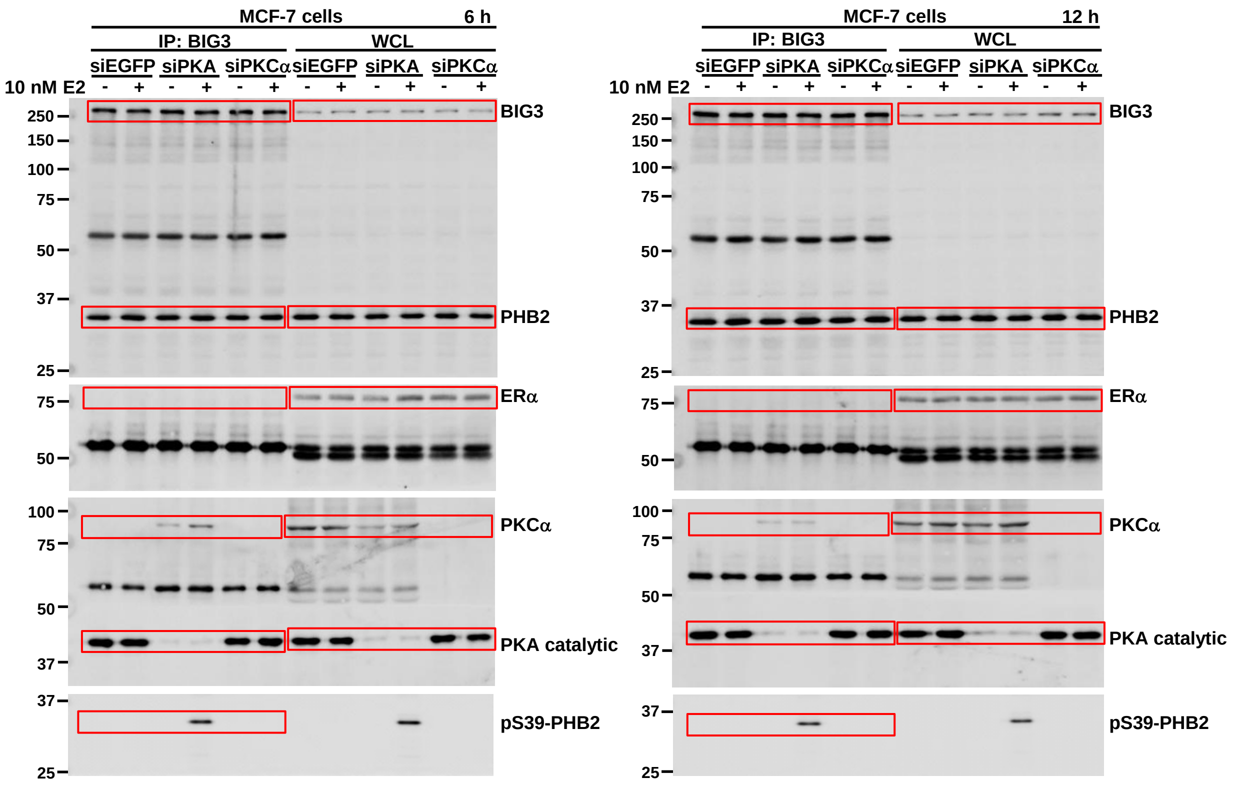

Continued on the following page

Uncropped images of Figure 3e

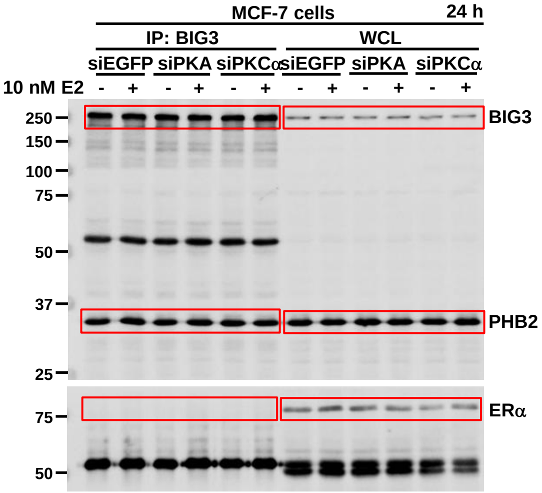

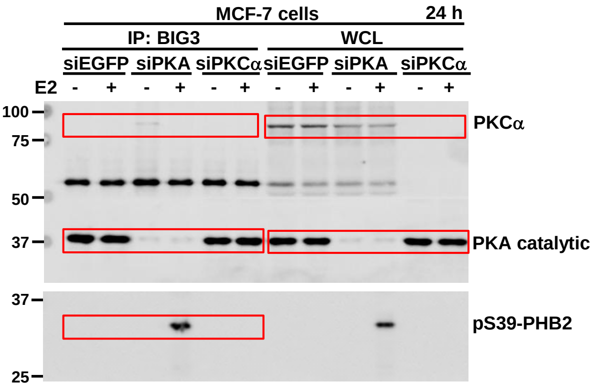

Uncropped images of Supplementary Figure 1a

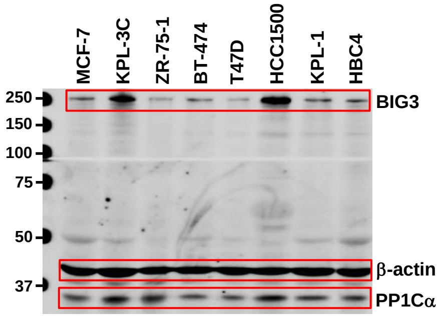

Uncropped images of Supplementary Figure 1b

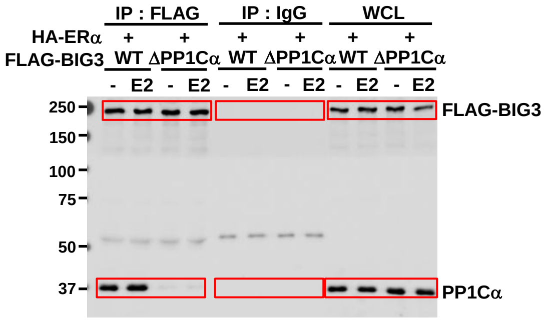

Uncropped images of Supplementary Figure 1c

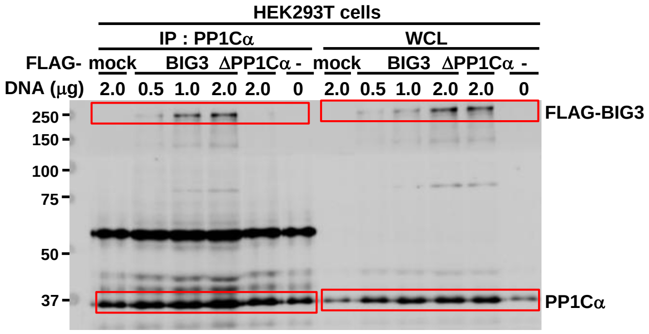

Uncropped images of Supplementary Figure 1d

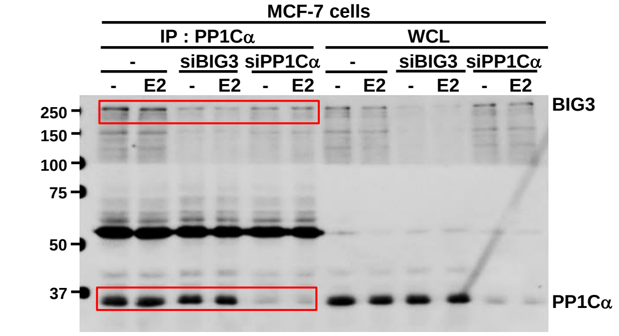

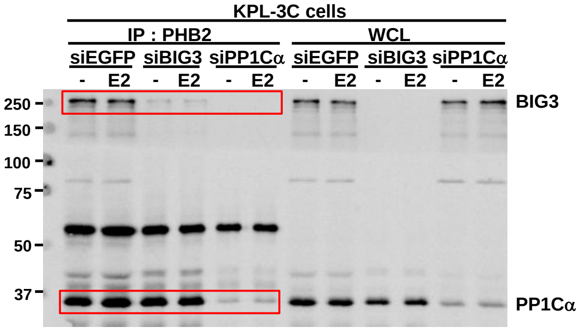

Continued on the following page

Uncropped images of Supplementary Figure 1f

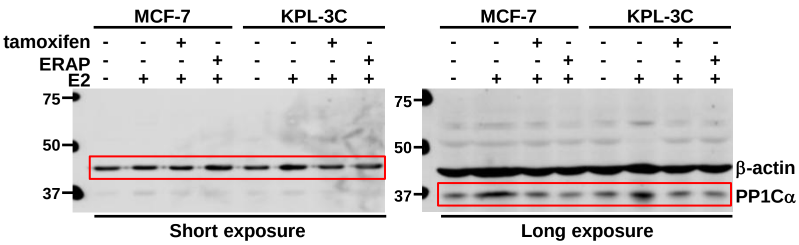

Uncropped images of Supplementary Figure 1h

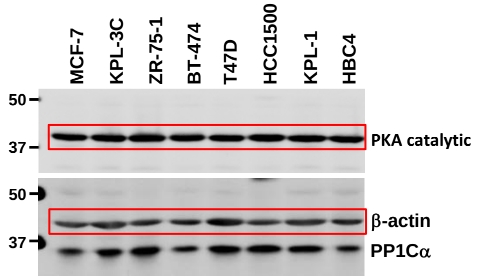

Uncropped images of Supplementary Figure 1m

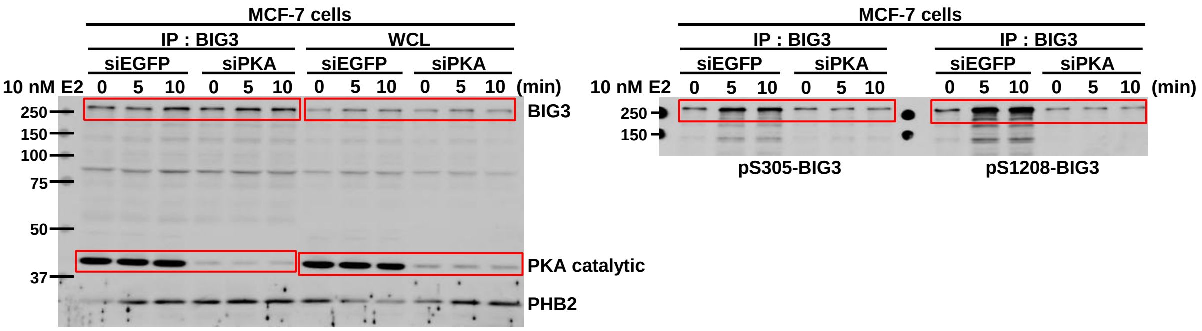

Uncropped images of Supplementary Figure 2a

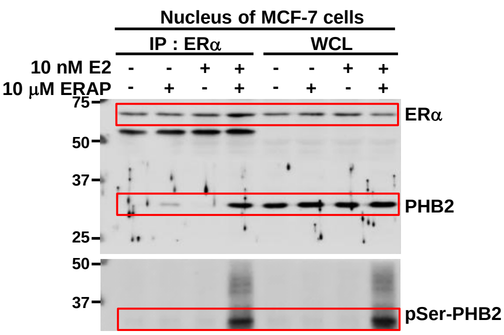

Uncropped images of Supplementary Figure 2b

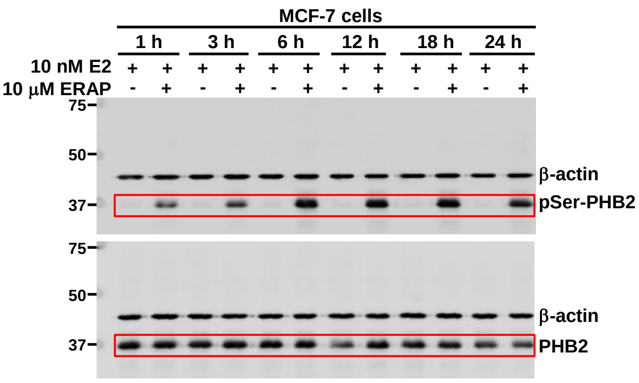

Continued on the following page

Uncropped images of Supplementary Figure 2c

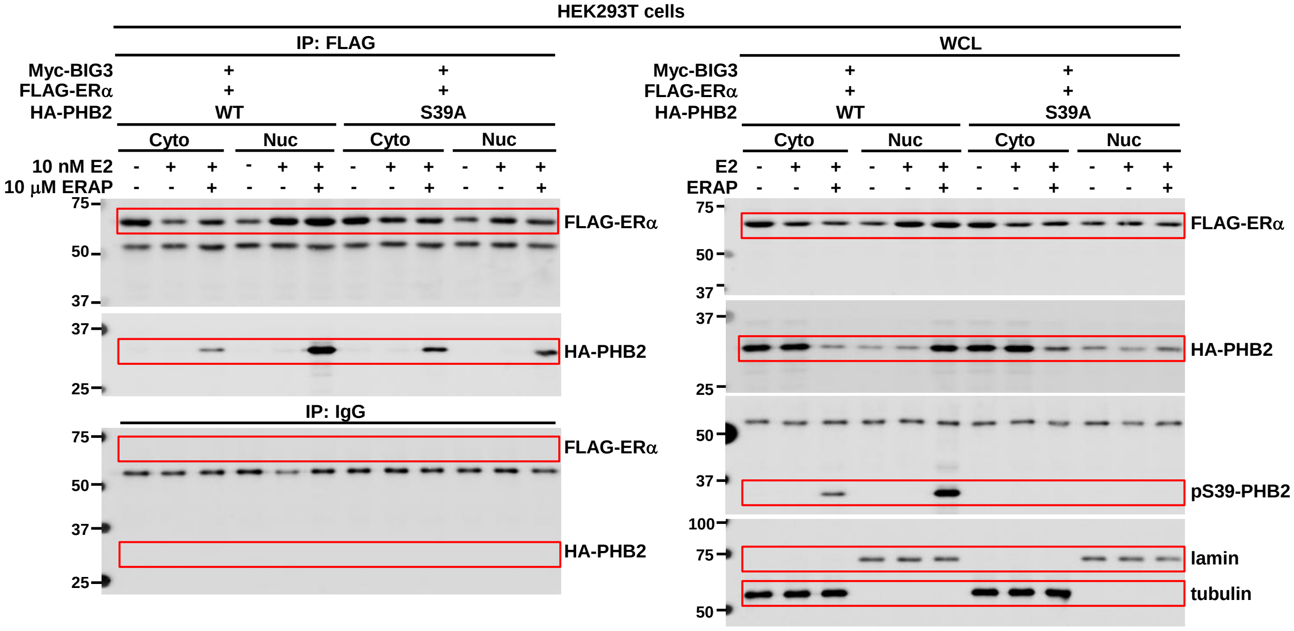

Uncropped images of Supplementary Figure 3a

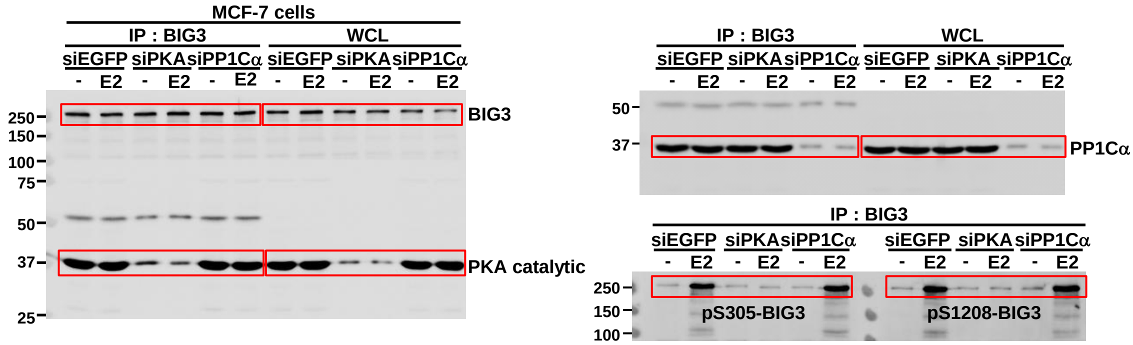

Uncropped images of Supplementary Figure 3b

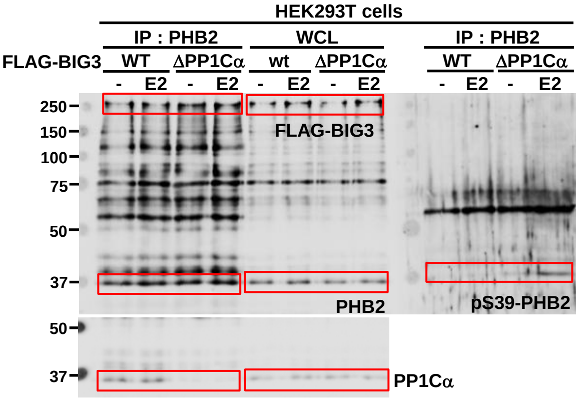

Uncropped images of Supplementary Figure 3c

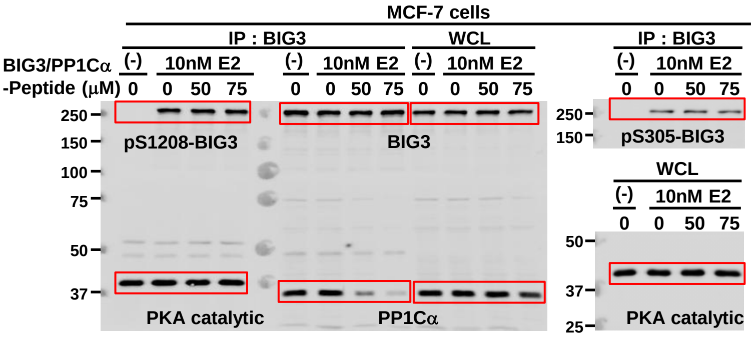

Continued on the following page

Uncropped images of Supplementary Figure 3d

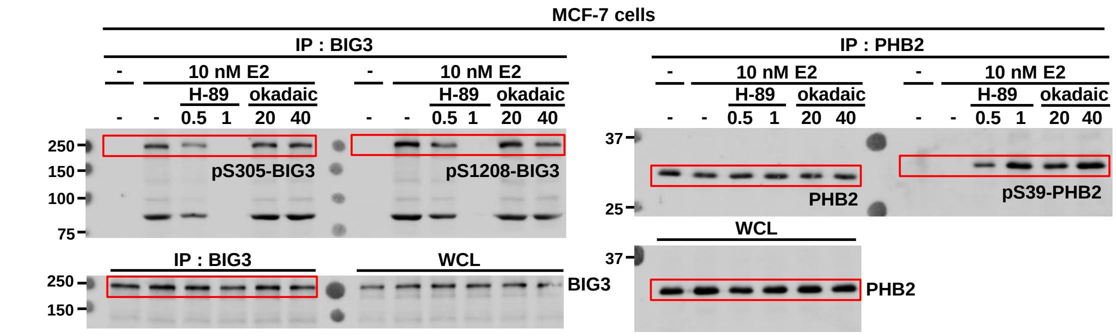

Uncropped images of Supplementary Figure 4b

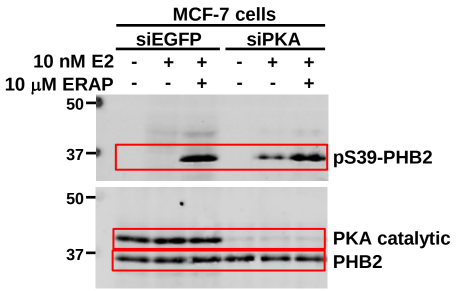

Uncropped images of Supplementary Figure 4d

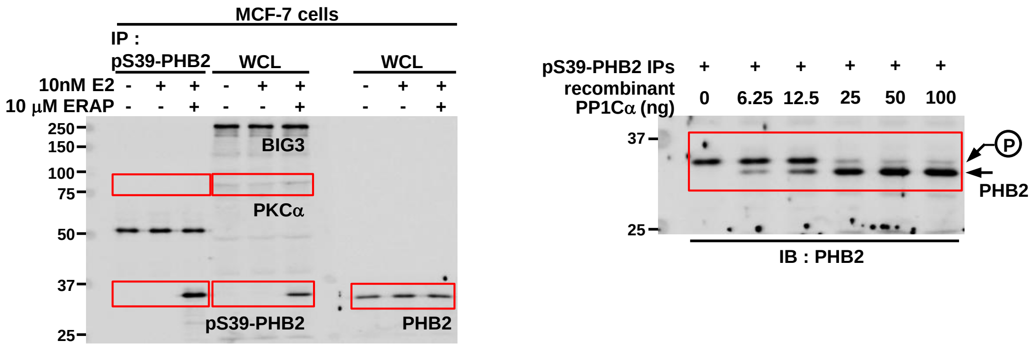

Supplementary Figure 6 | Full-length images of immunoblots. Uncropped images of scanned immunoblots in Figures and Supplementary figures with size marker indications (kDa)

Supplementary Table 1. Mascot Search Results MS/MS Fragmentation of GSGCSCTAPALSGPVAR (upper) and CWSLVAPH (lower) found in BIG3_HUMAN in Sprot

Score	Mr(calc)	Delta	Sequence	Site Analysis
57.6	1741.7165	-0.0212	GSGCSCTAPALSGPVAR	Propionamide C6, Carboxymethyl C4, Phospho S2; 45.13%
57.6	1741.7165	-0.0212	GSGCSCTAPALSGPVAR	Propionamide C4, Carboxymethyl C6, Phospho S2; 45.13%
47.6	1741.7165	-0.0212	GSGCSCTAPALSGPVAR	Propionamide C6, Carboxymethyl C4, Phospho S5; 4.45%
47.6	1741.7165	-0.0212	GSGCSCTAPALSGPVAR	Propionamide C4, Carboxymethyl C6, Phospho S5; 4.45%
37.1	1741.7165	-0.0212	GSGCSCTAPALSGPVAR	Propionamide C6, Carboxymethyl C4, Phospho T7; 0.40%
37.1	1741.7165	-0.0212	GSGCSCTAPALSGPVAR	Propionamide C4, Carboxymethyl C6, Phospho T7; 0.40%
20.6	1741.7052	-0.0100	SEQPGSILGPECASCK	
18.3	1741.7178	-0.0225	PNGTFSLHYFPYYG	
18.0	1741.7520	-0.0567	QNKQSSESAVSSTVNP	
18.0	1741.7520	-0.0567	QNKQSSESAVSSTVNP	

Score	Mr(calc)	Delta	Sequence
56.3	1062.4358	0.0069	CWSLVAPH
27.3	1062.4383	0.0043	GPGTQLPEGQ
21.5	1062.4747	-0.0321	GTATVLSTPH
21.5	1062.4747	-0.0321	GTATVLSTPH
20.4	1062.4035	0.0391	LSAVLSQEG
20.3	1062.4821	-0.0394	VSCPLPPVT
19.1	1062.4284	0.0142	GPDRFSAPH
19.0	1062.4747	-0.0321	AGPGTGLPGGLS
18.6	1062.3916	0.0511	YVCIPGCT
17.2	1062.4747	-0.0321	HDVVLVGGST

Supplementary Table 2. Clinical characteristic and results of immunohistochemistry of ERα-positive breast cancer specimens.

Case	BIG3 intensity (0-3)	PHB2 intensity (0-3)		DFI (year)	Recurrence	Age	Menopause	Tumour size	Lymph node metastasis	Stage
		Nucleus	Cytoplasm							
1	2	2	1	5.00	+	44	Pre	< 2 cm	n0	I
2	0	2	1	8.09		48	Post	2.1 - 5 cm	n0	I
7	1	2	2	6.48		44	Pre	< 2 cm	n0	IIA
9	2	3	1	10.00		69	Post	2.1 - 5 cm	n1	IIB
10	2	2	1	6.92	+	58	Post	2.1 - 5 cm	n1	II
12	2	0	1	7.17		63	Post	2.1 - 5 cm	n0	IIA
20	1	3	2	10.00		48	Pre	2.1 - 5 cm	n0	I
22	1	0	1	8.81		71	Post	< 2 cm	n0	IIA
29	0	0	0	7.02		82	Post	< 2 cm	n0	IIA
30	3	3	1	5.83	+	47	Pre	2.1 - 5 cm	n0	IIA
32	1	0	0	6.84	+	70	Post	< 2 cm	n1	IIB
35	1	2	1	4.51	+	55	Post	< 2 cm	n0	II
36	1	3	1	7.22		69	Post	2.1 - 5 cm	n0	IIA
37	1	3	1	8.05		58	Post	< 2 cm	n0	I
39	3	0	0	6.73		42	Pre	2.1 - 5 cm	n0	I
41	1	3	1	9.48		45	Pre	2.1 - 5 cm	n0	I
43	3	0	1	8.24		51	Post	< 2 cm	n0	I
44	3	1	2	5.75		58	Post	2.1 - 5 cm	n0	I
45	2	3	2	8.01		48	Pre	2.1 - 5 cm	n1	I
46	2	0	0	5.62		46	Pre	2.1 - 5 cm	n0	I
47	2	0	0	5.54		40	Pre	2.1 - 5 cm	n0	I
48	3	1	0	1.17	+	51	Post	2.1 - 5 cm	n0	IIA
49	1	3	1	10.00		49	Pre	< 2 cm	n0	I
50	2	2	1	7.05		55	Post	< 2 cm	n0	I
51	2	0	1	6.38		44	Pre	< 2 cm	n0	I
55	2	0	1	6.13		55	Post	2.1 - 5 cm	n0	I
57	0	1	1	7.06		71	Post	2.1 - 5 cm	n1	IIA
58	2	0	0	5.83		46	Pre	< 2 cm	n0	I
61	2	3	1	9.06		67	Post	< 2 cm	n0	IIA
62	2	3	2	9.98		48	Pre	< 2 cm	n1	I
70	2	3	1	10.00		51	Post	2.1 - 5 cm	n0	I
73	2	3	2	10.00		59	Post	< 2 cm	n0	I
74	3	0	0	7.11	+	55	Post	< 2 cm	n1	I
76	2	0	1	5.62		55	Post	< 2 cm	n0	I
77	3	0	1	6.21	+	63	Post	< 2 cm	n0	I
78	1	0	0	6.08		72	Post	< 2 cm	n0	I
79	0	3	2	5.57		54	Post	2.1 - 5 cm	n0	II
82	3	0	0	9.10	+	45	Pre	2.1 - 5 cm	n0	II
85	1	3	1	7.17		50	Pre	2.1 - 5 cm	n1	II
86	2	3	0	6.99		50	Pre	< 2 cm	n0	I
88	1	3	2	9.81		46	Pre	< 2 cm	n0	I
89	0	2	1	3.00		52	Pre	2.1 - 5 cm	n0	II
92	3	0	0	10.00	+	40	Pre	2.1 - 5 cm	n0	II
93	3	0	1	5.18	+	49	Pre	< 2 cm	n0	I
95	2	0	0	4.99		58	Post	< 2 cm	n0	I
96	2	0	0	4.00		78	Post	2.1 - 5 cm	n0	II
100	3	0	0	5.14	+	46	Pre	2.1 - 5 cm	n0	II
101	2	0	0	3.08		52	Pre	< 2 cm	n0	I
102	3	0	0	5.66		44	Pre	< 2 cm	n0	I
103	2	0	1	4.98		58	Post	< 2 cm	n0	I
117	1	0	0	4.49		48	Pre	< 2 cm	n0	I
118	1	1	0	4.68		64	Post	< 2 cm	n1	II
120	2	0	0	6.63	+	57	Post	< 2 cm	n0	II
122	2	1	1	3.12		53	Post	2.1 - 5 cm	n0	I
124	0	3	0	10.00		39	Pre	2.1 - 5 cm	n0	I
125	2	0	0	5.20		70	Post	< 2 cm	n0	II
129	2	0	0	2.42	+	68	Post	2.1 - 5 cm	n0	II
135	2	0	1	4.67		70	Post	< 2 cm	n0	I
136	1	0	0	5.38		47	Post	< 2 cm	n0	I
138	1	0	0	6.75		51	Pre	< 2 cm	n0	IIA
145	2	0	0	4.80		56	Post	< 2 cm	n0	I

Continued on the following page

146	1	3	2	9.99		58	Post	< 2 cm	n0	I
155	2	0	0	4.66		68	Post	2.1 - 5 cm	n0	II
156	0	2	1	9.05		46	Pre	< 2 cm	n0	I
159	3	0	0	2.25	+	40	Pre	2.1 - 5 cm	n0	II
161	1	1	1	5.43		48	Pre	2.1 - 5 cm	n0	I
163	1	3	1	9.34		49	Pre	< 2 cm	n0	I
168	1	3	1	7.81		51	Post	2.1 - 5 cm	n0	II
170	0	2	2	8.08		63	Post	2.1 - 5 cm	n1	II
174	1	0	1	5.72		43	Pre	2.1 - 5 cm	n0	II
176	3	3	2	5.51		80	Post	2.1 - 5 cm	n0	II
180	3	0	0	5.00		78	Post	2.1 - 5 cm	n0	II
181	2	0	1	5.66		70	Post	< 2 cm	n0	I
182	3	0	0	5.60		68	Post	< 2 cm	n0	I
187	1	3	2	7.73		58	Post	< 2 cm	n0	I
192	2	3	1	8.04		71	Post	2.1 - 5 cm	n0	II
193	3	0	0	5.37		46	Pre	2.1 - 5 cm	n0	II
195	0	3	0	7.96		49	Post	< 2 cm	n0	I
198	2	0	0	4.93		54	Post	< 2 cm	n0	I
203	1	0	1	6.68		50	Pre	< 2 cm	n0	I
205	1	1	1	5.26		44	Pre	2.1 - 5 cm	n0	II
208	1	3	2	10.00		47	Pre	< 2 cm	n1	I

Supplementary Table 3. The sequences of each primer set.

Genes	Sequence	Purpose
BIG3	5’-CGGAATTCATGGAAGAAATCCTGAGGA AGC-3′ (forward)<br>5′-ATAGTTTAGCGGCCGCACAATGATGTCATAGACACGG-3′ (reverse)	Cloning
BIG3 mutant (T162A)	5’-GTGCGGGCAGCCCTCAGTCAA-3′ (forward)<br>5′-TTGACTGAGGGCTGCCCGCAC-3′ (reverse)	Cloning
BIG3 mutant (S305A)	5’-TCAGGCTGCGCCTGCACTGCG-3′ (forward)<br>5′-CGCAGTGCAGGCGCAGCCTGA-3′ (reverse)	Cloning
BIG3 mutant (S305E)	5’-GGCCGAGGAGAAGGCTGCTCC-3′ (forward)<br>5’-GGAGCAGCCTTCTCCTCGGCC-3′ (reverse)	Cloning
BIG3 mutant (S689A)	5’-CGGCTCCTGGCCCTCTCCAAT-3′ (forward)<br>5′-ATTGGAGAGGGCCAGGAGCCG-3′ (reverse)	Cloning
BIG3 mutant (S925A)	5’-GCACGGCTGGCCTGCGCTCTA-3′ (forward)<br>5′-TAGAGCGCAGGCCAGCCGTGC-3′ (reverse)	Cloning
BIG3 mutant (S1208A)	5’-CGCTGCTGGGCCCTTGTGGCC-3′ (forward)<br>5′-GGCCACAAGGGCCCAGCAGCG-3′ (reverse)	Cloning
BIG3 mutant (S1208E)	5’-CGCTGCTGGGAACTTGTGGCC-3′ (forward)<br>5’-GGCCACAAGTTCCCAGCAGCG-3′ (reverse)	Cloning
BIG3 mutant (S1763A)	5’-AGATACATCGCCATGCAGAAC-3′ (forward)<br>5′-GTTCTGCATGGCGATGTATCT-3′ (reverse)	Cloning
PHB2	5′-CGGAATTCCAGACCGTGCATCATGGCCCAGAACTTGAAGGA-3′ (forward)<br>5′-CCGCTCGAGTTTCTTACCCTTGATGAGGCTGT-3′ (reverse)	Cloning
PHB2 (S39A)	5′-GTGCGCGAAGCCGTGTTCACC-3′ (forward)<br>5′-GGTGAACACGGCTTCGCGCAC-3′ (reverse)	Cloning
PKCα	5’-CCCAAGAATGAAAGCAAGCA-3’ (forward)<br>5’-CCGAAACTCCAAAGGAAAGG-3’ (reverse)	Real-time PCR
PKCε	5’-TCAAGCAGCACCCATTCTTC-3’ (forward)<br>5’-TCAGGGCATCAGGTCTTCAC-3’ (reverse)	Real-time PCR
CAMK2	5’-TCAGGGCATCAGGTCTTCAC-3’ (forward)<br>5’-GGGGAGAGAGGCAATGAAGA-3’ (reverse)	Real-time PCR
β2-microglobulin	5’-AACTTAGAGGTGGGGAGCAG-3’ (forward)<br>5’-CACAACCATGCCTTACTTTATC-3’ (reverse)	Real-time PCR

The underlines indicate the recognition sites of restriction enzymes.
Double-underlines indicate the mutation sites.
